# Supplementary material for: Global search metaheuristics for neural mass model calibration
Source: Imaging Neurosci (Camb). 2026 Jun 5;4:IMAG.a.1249. doi: 10.1162/IMAG.a.1249 (PMC13245216; doi:10.1162/IMAG.a.1249)
Supplement: Supplementary Material [file IMAG.a.1249_supp.pdf]

Supplemental

Global search metaheuristics for neural mass model calibration

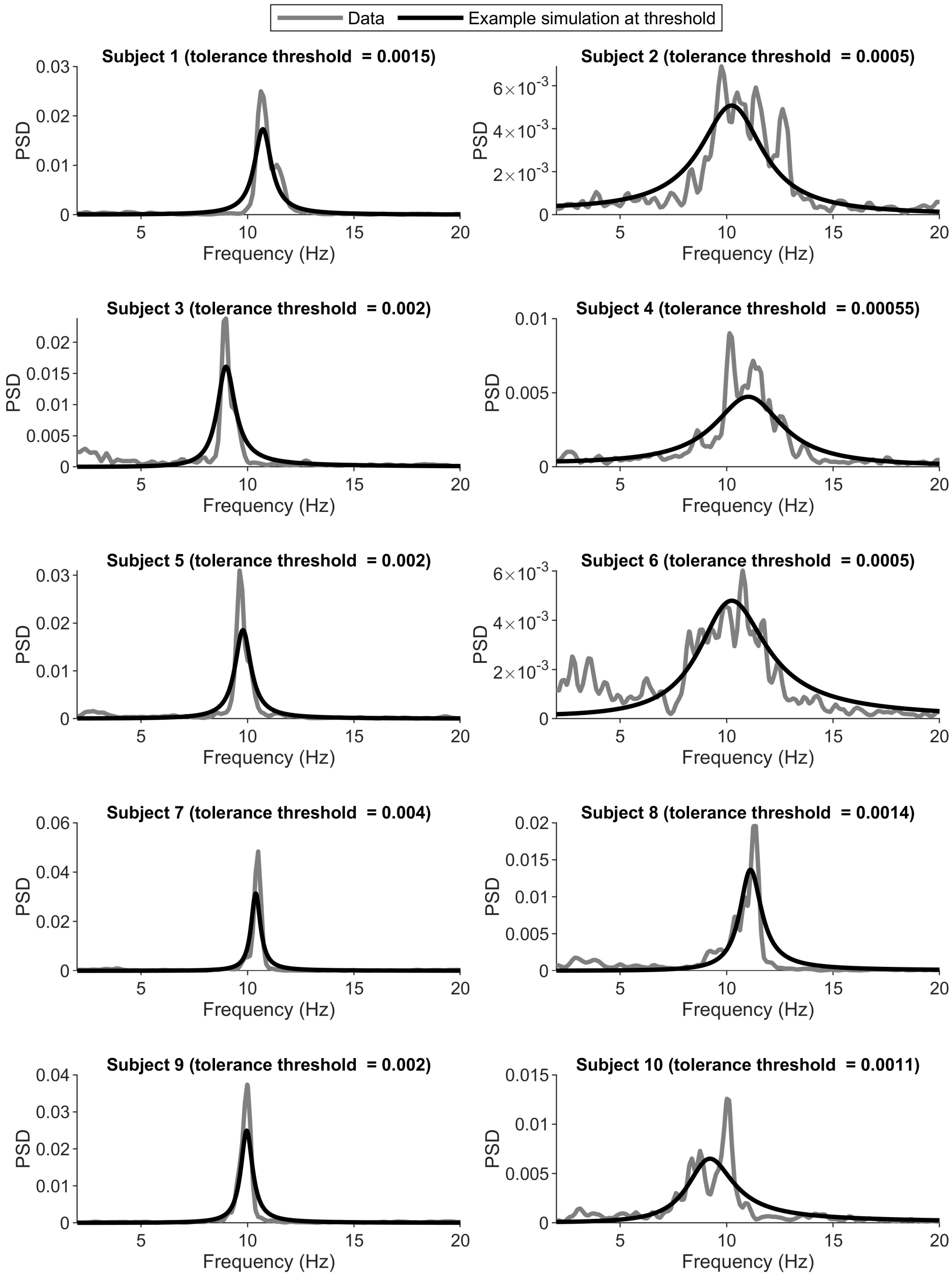

Figure S1: **Data PSD and example model PSD at cost tolerance threshold.** Each subplot shows a different subject. The model PSDs show an example simulation at the cusp of the tolerance threshold (as defined for each subject in the corresponding subplot title). Simulations under this cost are regarded as a sufficiently good fit to the data.

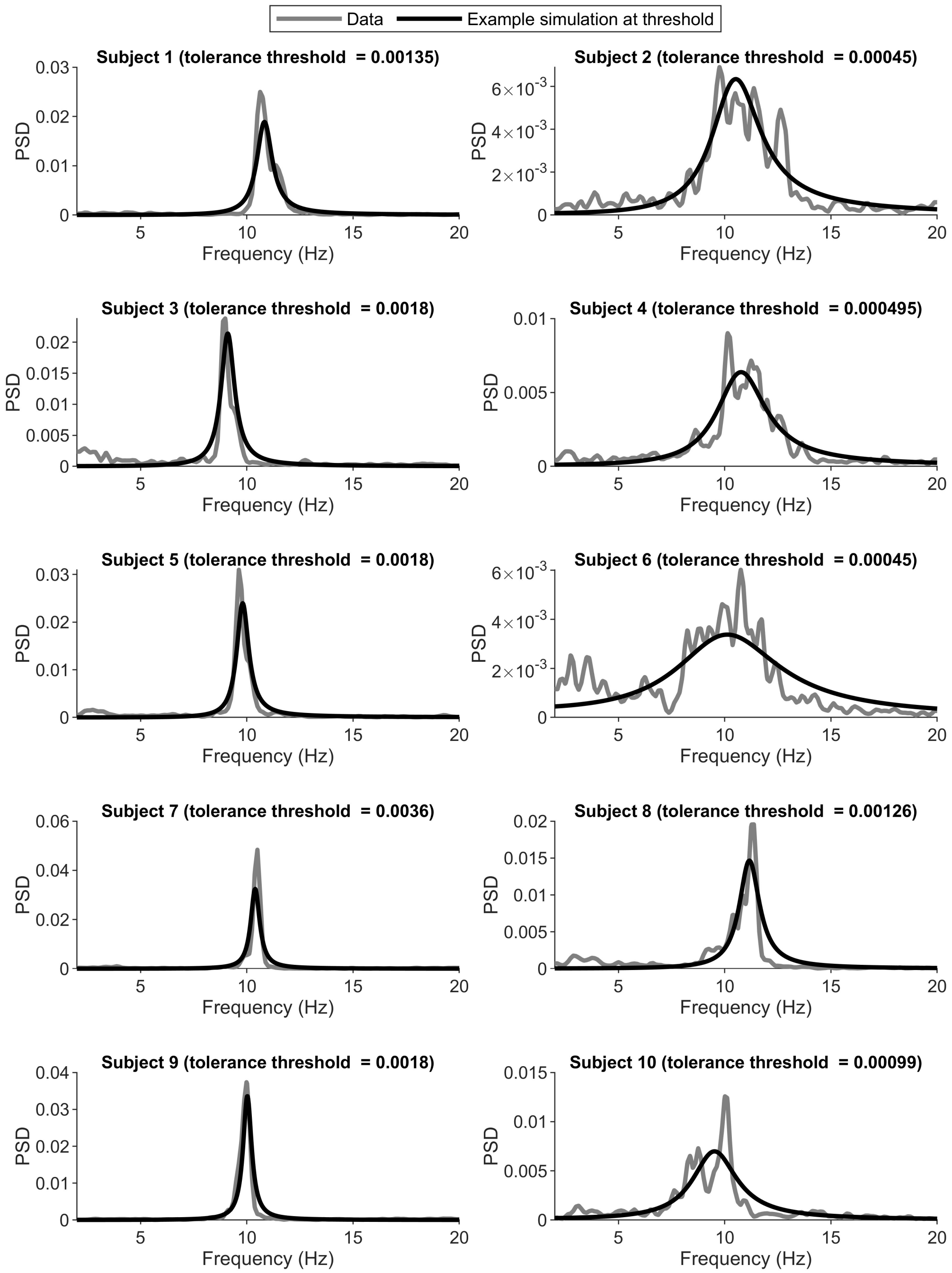

Figure S2: **Data PSD and example model PSD at cost tolerance threshold 2.** Each subplot shows a different subject. The model PSDs show an example simulation at the cusp of the tolerance threshold (as defined for each subject in the corresponding subplot title). Simulations under this cost are regarded as a sufficiently good fit to the data. This example shows a tolerance 10% stricter than Fig. S1.

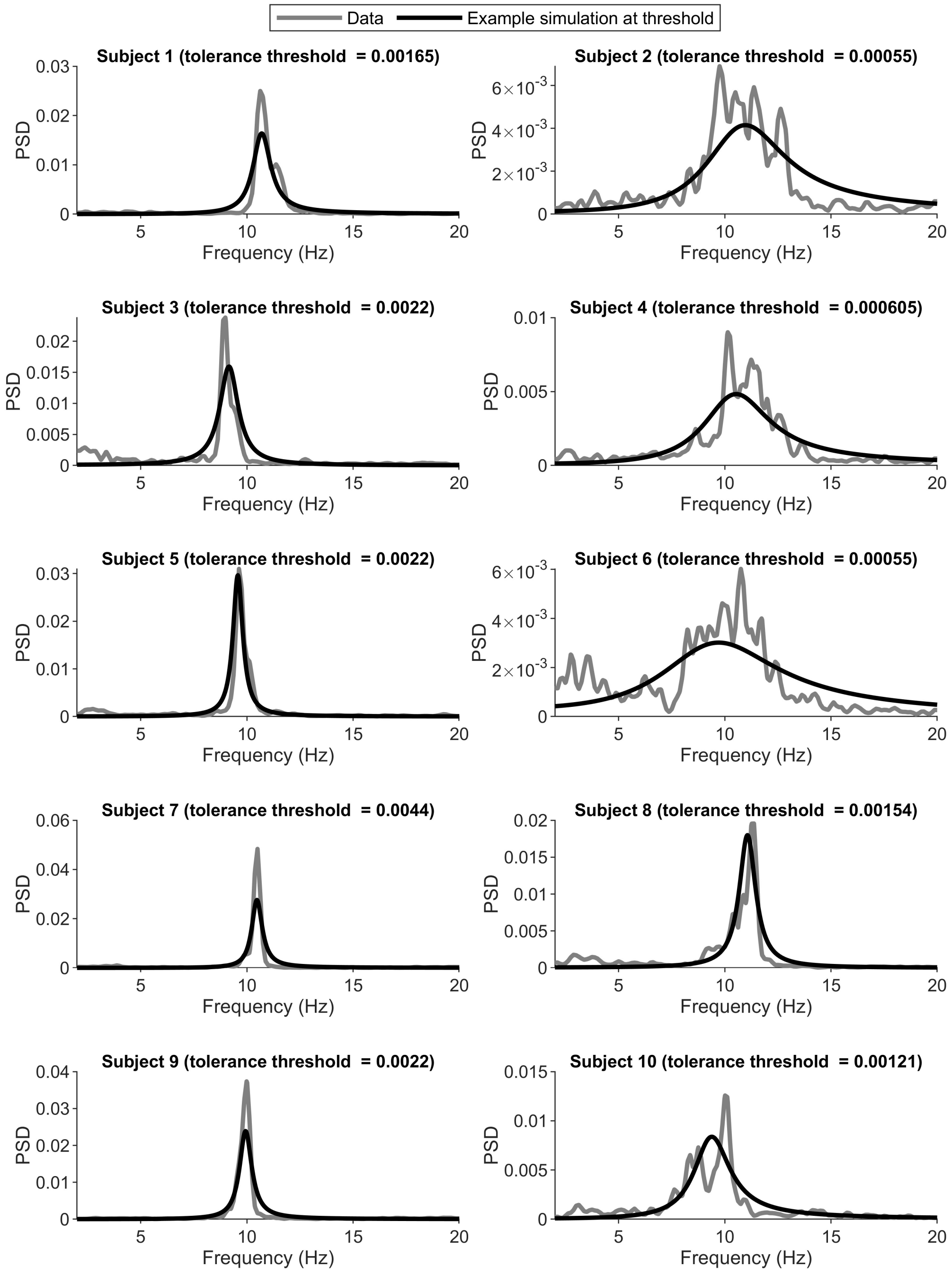

Figure S3: **Data PSD and example model PSD at cost tolerance threshold 3.** Each subplot shows a different subject. The model PSDs show an example simulation at the cusp of the tolerance threshold (as defined for each subject in the corresponding subplot title). Simulations under this cost are regarded as a sufficiently good fit to the data. This example shows a tolerance 10% more lenient than Fig. S1.

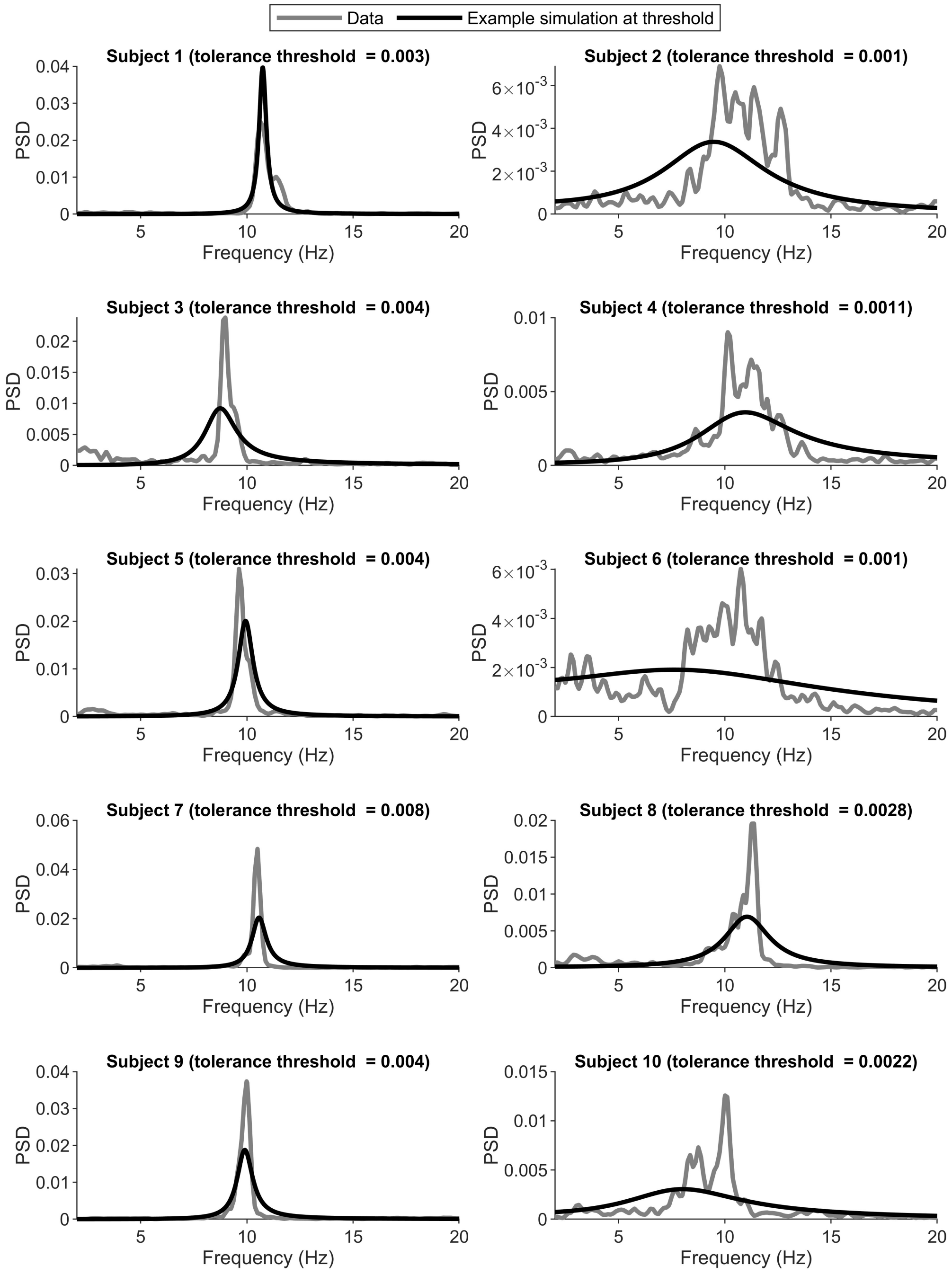

Figure S4: **Data PSD and example model PSD at cost tolerance threshold 4.** Each subplot shows a different subject. The model PSDs show an example simulation at the cusp of the tolerance threshold (as defined for each subject in the corresponding subplot title). Simulations under this cost are regarded as a sufficiently good fit to the data. This example shows a tolerance 100% more lenient than Fig. S1.

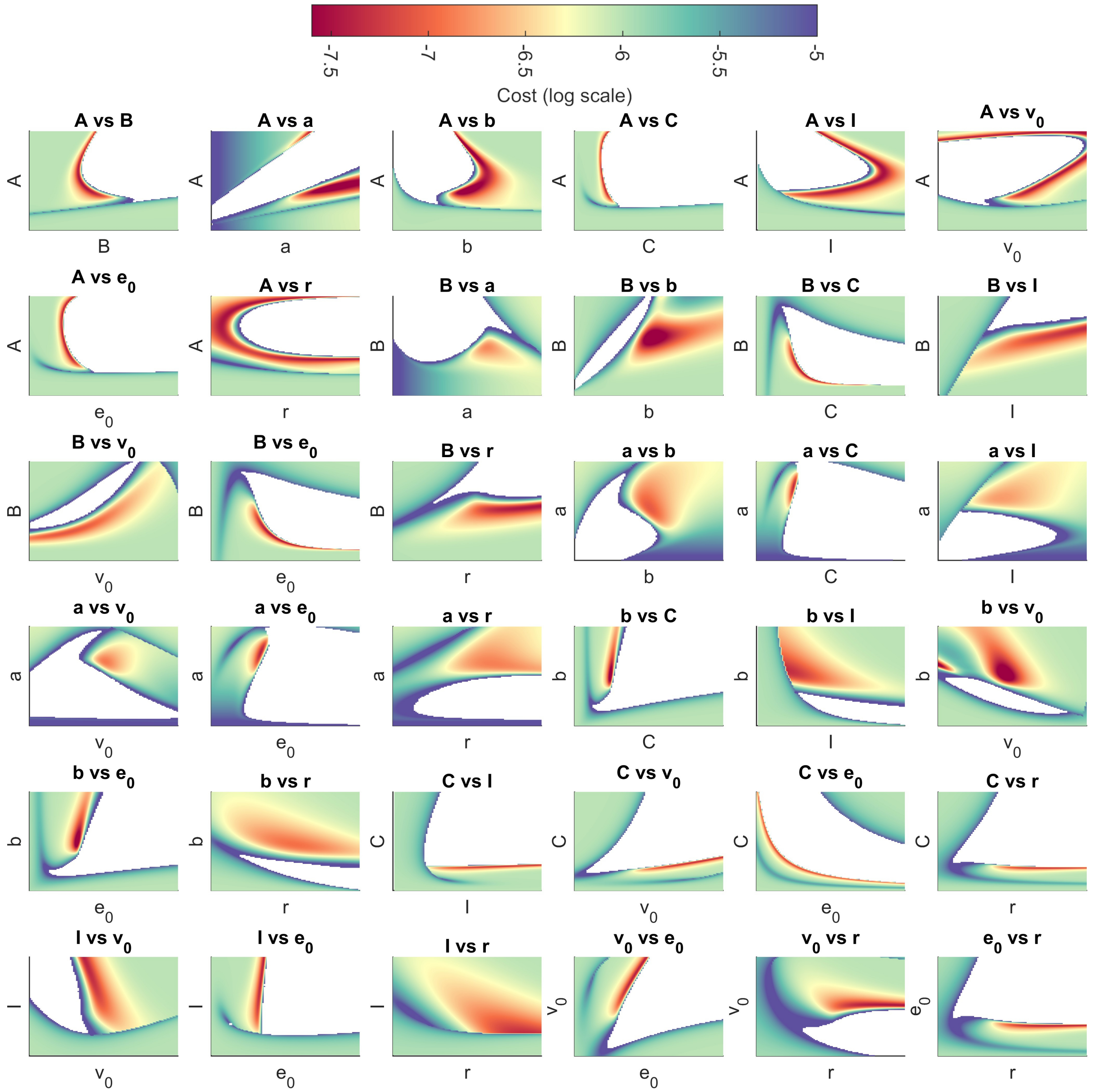

Figure S5: **Cost landscape of the Jansen and Rit model across pairwise model parameter combinations.** Subplot titles (and axes) denote the parameter combinations, with other model parameters set to their typical values (see Table 1 in the Manuscript). Axis limits are set to the bounds defined in Table 1 of the Manuscript. The cost (on a log scale and measured by the sum of squared error) shows how well the model at the given parameter combination simulates the data PSD. These figures were generated from comparing model simulations to the PSD estimated from the EEG of subject 2. The white region shows an area where no stable fixed points were found, and hence the cost could not be calculated from the linearised equations in this region.

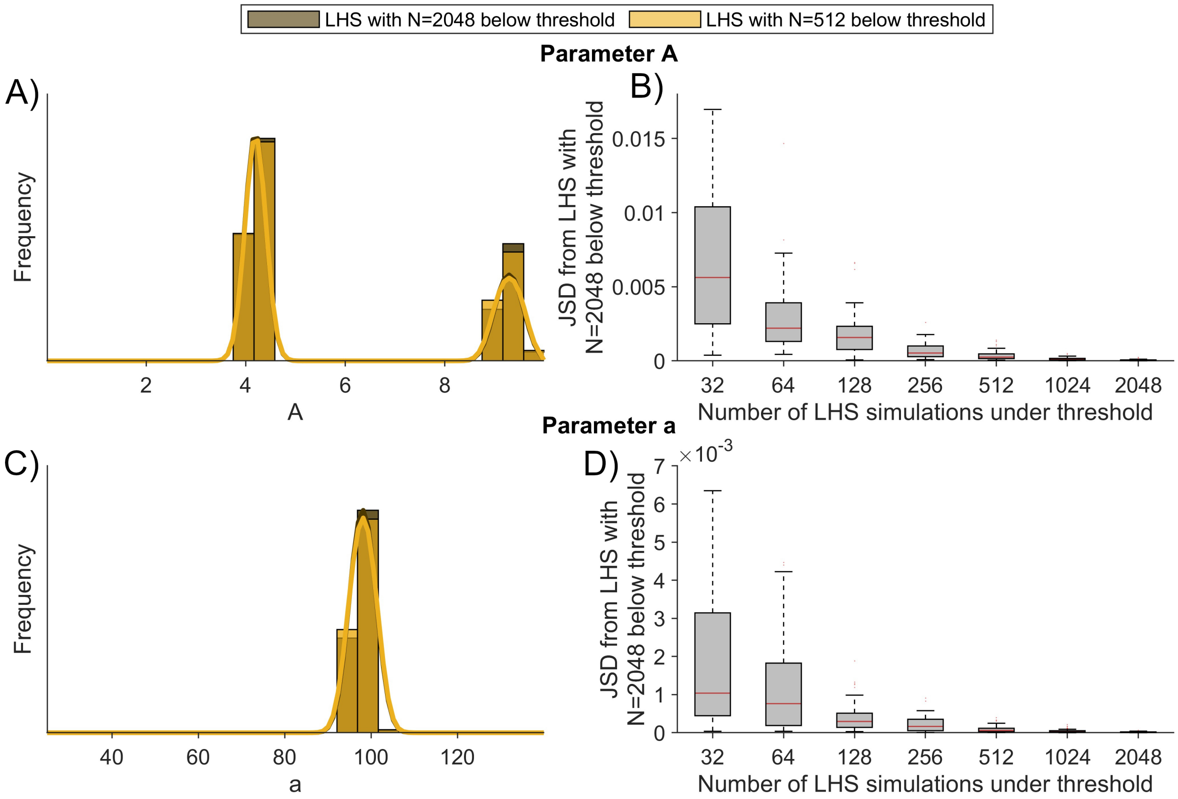

Figure S6: **Comparison of LHS resolutions for subject 1 in 2D.** A) Marginal density approximations obtained for parameter A from subject 1 using different LHS resolutions (see legend). B) JSD from the finest LHS resolution (N=2048 points under tolerance threshold) to different resolutions. C) and D) show the same as A) and B), respectively, but for parameter a. For each parameter, the x-axis limits are set to the parameter's bounds, and the histograms give the observed frequencies, shown superimposed with a kernel density estimate. The actual LHS size used was N=512, which provided an acceptable resolution.

**ABC tolerance schedule**	**Number of generations**	**Mean acceptance rate**
$[f_1]$	1	[0.00190]
$[5f_1,1.5f_1,f_1]$	3	[0.749,0.0342,0.00713]
$[5f_1,2f_1,1.5f_1,f_1]$	4	[0.742,0.1840.0525,0.0131]
$[5f_1,3f_1,2f_1,1.75f_1,1.5f_1,1.25f_1,f_1]$	7	[0.735,0.650,0.210,0.144,0.0744,0.0533,0.0280]

Table S1: **ABC tolerance schedules and acceptance rates.** Mean (across subjects) acceptance rates for different ABC tolerance schedules, as analysed in 3 dimensions and shown in Fig. S8. Here, the tradeoff between how strict the tolerance schedule is and the acceptance rate can be seen. f_1 represents the final tolerance used. The tolerance schedule with 4 generations is ultimately chosen as the tolerance used for ABC throughout the paper.

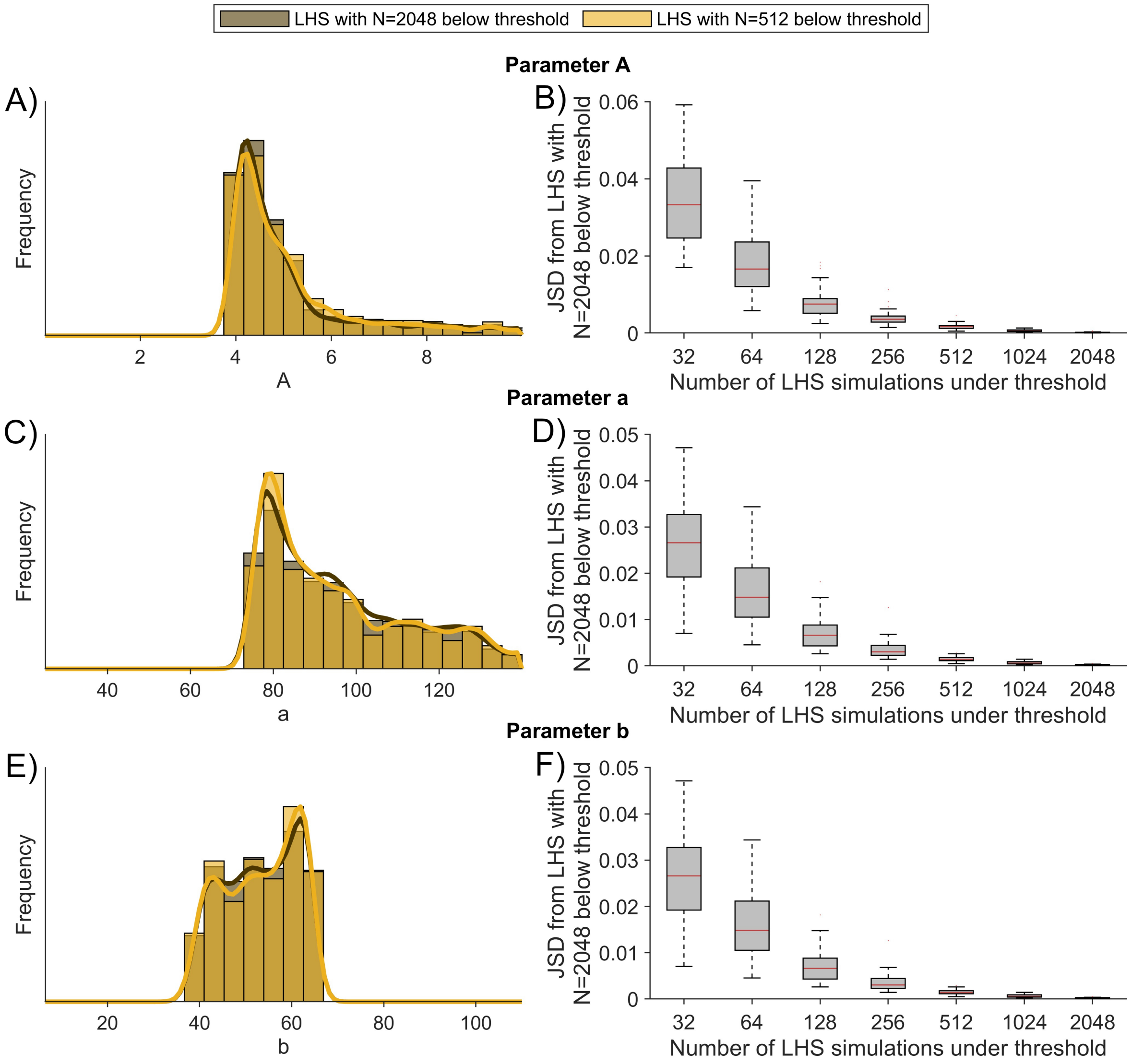

Figure S7: **Comparison of LHS resolutions for subject 1 in 3D.** A) Marginal density approximations obtained for parameter A from subject 1 using different LHS resolutions (see legend). B) JSD from the finest LHS resolution (N=2048 points under tolerance threshold) to different resolutions. C) and D) show the same as A) and B), respectively, but for parameter a. E) and F) show the same as A) and B), respectively, but for parameter b. For each parameter, the x-axis limits are set to the parameter's bounds, and the histograms give the observed frequencies, shown superimposed with a kernel density estimate. The actual LHS size used was N=512, which provided an acceptable resolution.

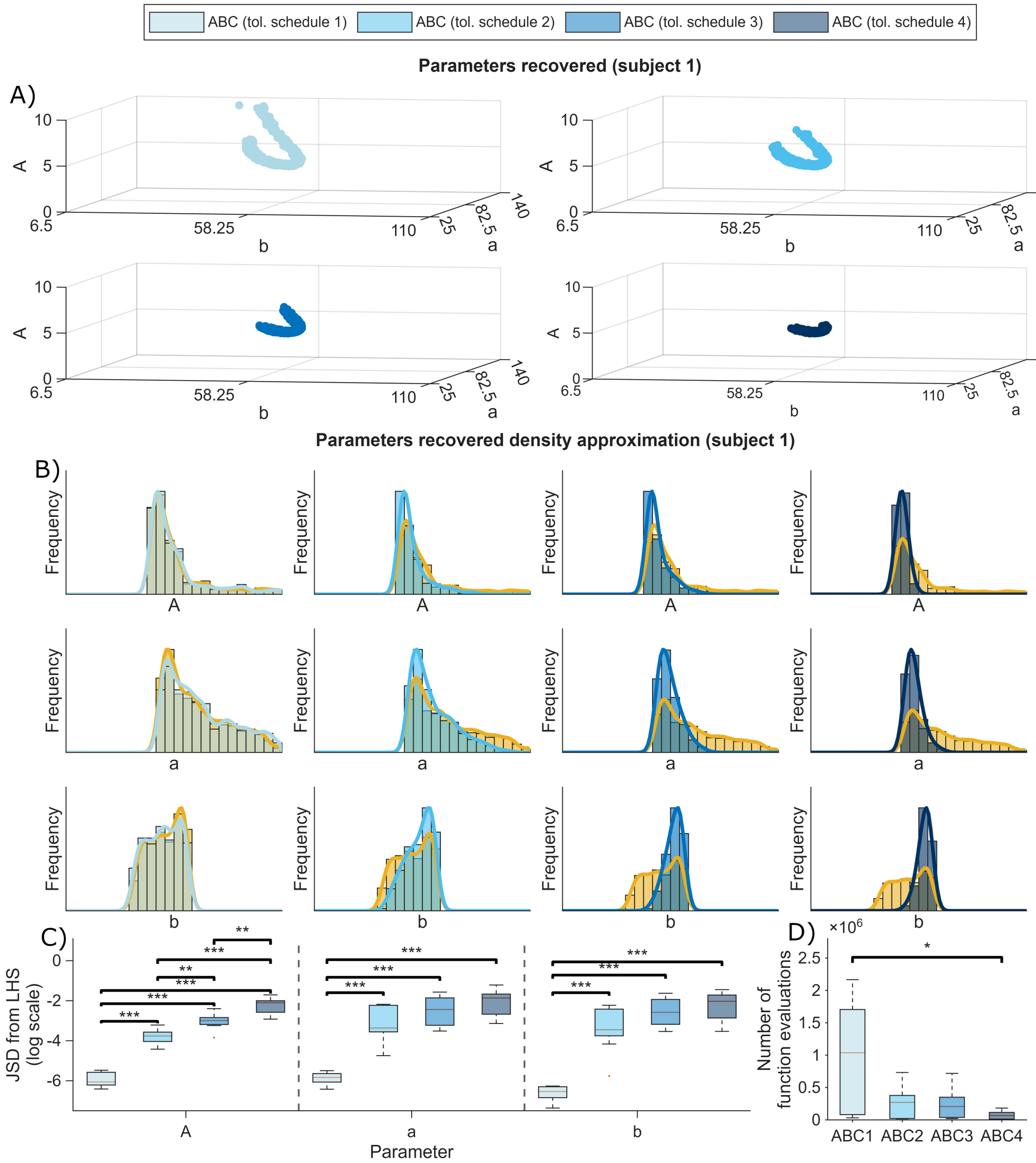

Figure S8: **Comparison of algorithms for model calibration in 3 dimensions using different ABC tolerance schedules.** A) Model parameters recovered for subject 1 from ABC with different tolerance schedules. See Table S1 for a list of the 4 tolerance schedules used. Colours indicate regions where feasible parameters were found. B) Marginal density approximations obtained using each tolerance schedule to recover parameters from subject 1. For each parameter, the x-axis limits are set to the parameter's bounds, and the histograms give the observed frequencies, shown superimposed with a kernel density estimate. Each tolerance schedule tested is shown superimposed on the approximation obtained from the LHS, which represents the ground truth. In each case, the distributions consist of N=512 points and the model was calibrated by varying 3 parameters (A, a and b) within their bounded constraints, with other model parameters fixed to their typical values (see Table 1 in the manuscript). C) JSD between the parameter distributions obtained from the LHS compared to the parameter distributions obtained from each method (log scale). D) The number of function evaluations used to execute each method. *$p < 0.05$ using a Mann-Whitney U test with Bonferroni correction.

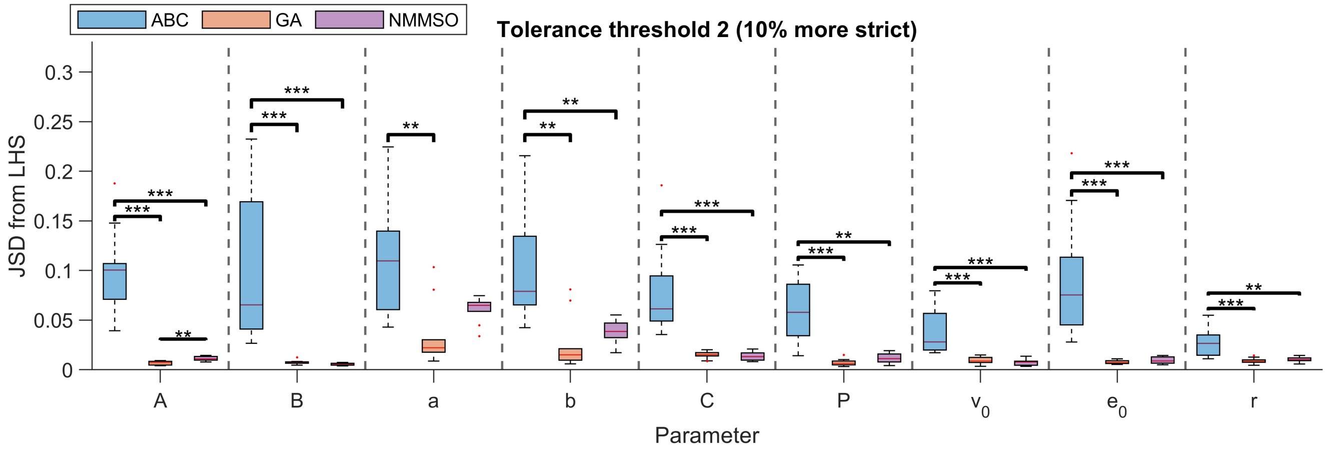

Figure S9: **JSD from tolerance thresholds defined in Fig. S2.** The JSD between parameter distributions obtained from an LHS and each algorithm is shown (see legend). These were obtained from varying all 9 parameters in the Jansen and Rit model and using the tolerance threshold defined in Fig. S2 (10% stricter). ***$p < 0.001$, **$p < 0.01$, *$p < 0.05$ using a Mann-Whitney U test with Bonferroni correction.

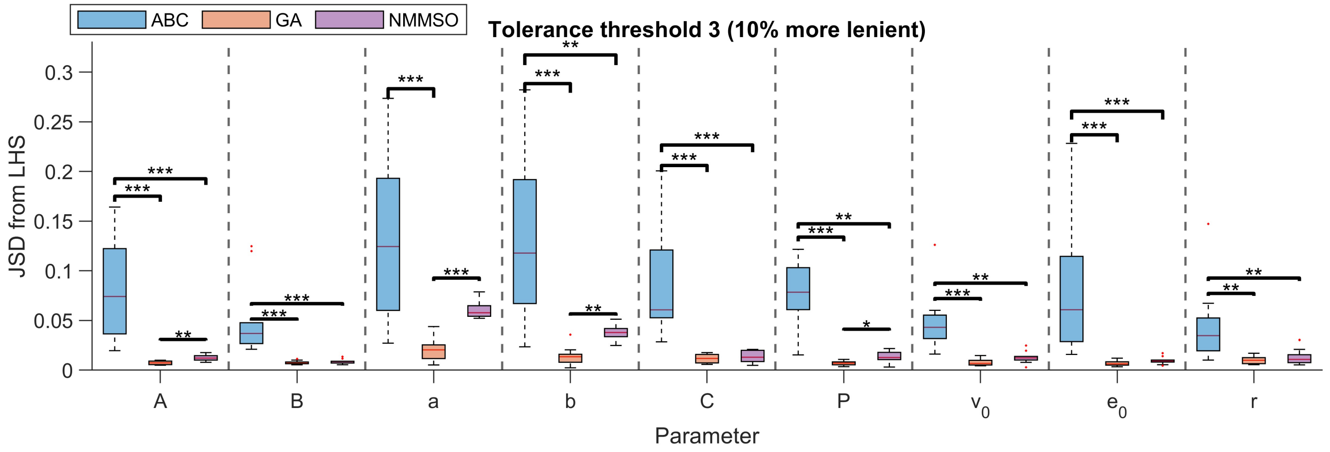

Figure S10: **JSD from tolerance thresholds defined in Fig. S3.** The JSD between parameter distributions obtained from an LHS and each algorithm is shown (see legend). These were obtained from varying all 9 parameters in the Jansen and Rit model and using the tolerance threshold defined in Fig. S3 (10% more lenient). ***$p < 0.001$, **$p < 0.01$, *$p < 0.05$ using a Mann-Whitney U test with Bonferroni correction.

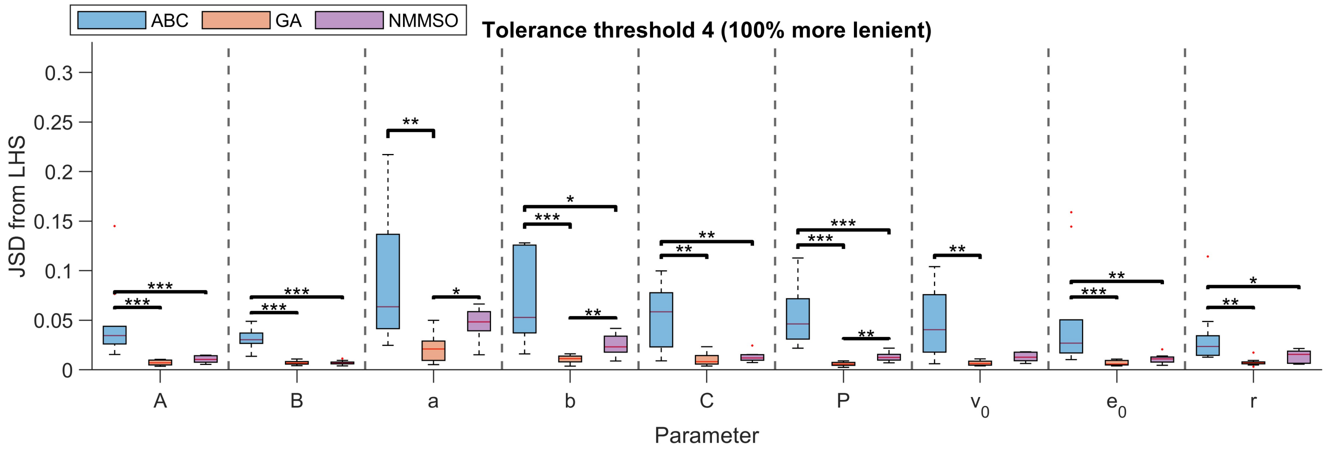

Figure S11: **JSD from tolerance thresholds defined in Fig. S4.** The JSD between parameter distributions obtained from an LHS and each algorithm is shown (see legend). These were obtained from varying all 9 parameters in the Jansen and Rit model and using the tolerance threshold defined in Fig. S4 (100% more lenient). ***$p < 0.001$, **$p < 0.01$, *$p < 0.05$ using a Mann-Whitney U test with Bonferroni correction.

Simulating the full nonlinear model

To simulate the full nonlinear model, we used the Euler–Maruyama method with zero initial conditions. A time step of 0.3 ms was used to ensure accurate convergence. Each model output was calculated for a total of 20 s to match the data epoch length. To account for transient dynamics, a further 5 s was calculated at the start of the model simulation and then removed. We used a low noise variance to simulate the model (noise variance =1). The PSD was computed from this time series using Welch's method (Welch, 1967), in an identical way as implemented for the data (see Manuscript for details). We repeated each model simulation 10 times and took the mean PSD across these repeats to generate an accurate PSD.

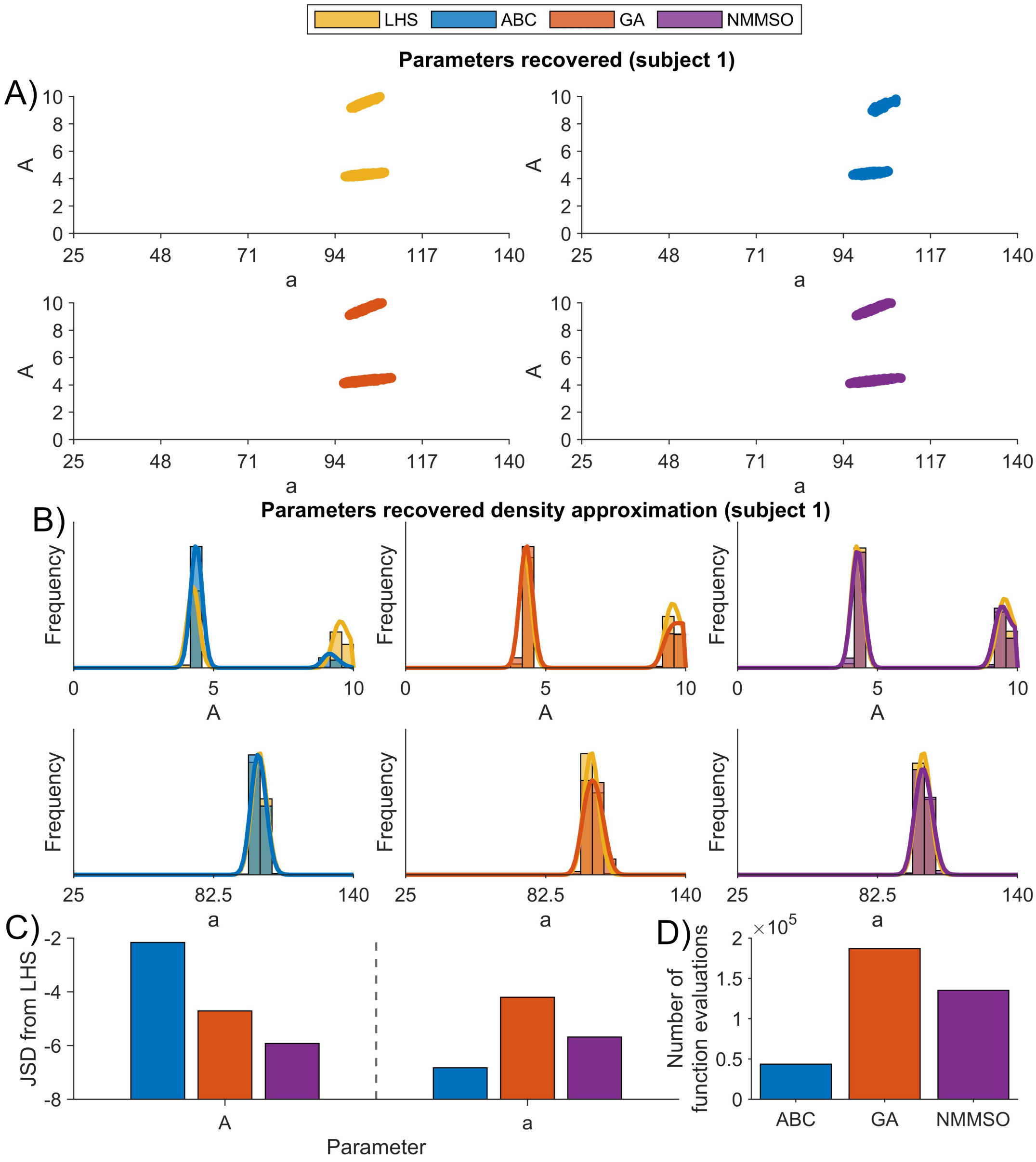

Figure S12: **Comparison of algorithms for model calibration in 2 dimensions using the full nonlinear model.** A) Model parameters recovered from the LHS (top left), ABC (top right), GA (bottom left) and NMMSO (bottom right) for subject 1. Colours indicate regions where feasible parameters were found. B) Marginal density approximations obtained using each algorithm to recover parameters from the EEG data of subject 1. For each parameter, the x-axis limits are set to the parameter's bounds, and the histograms give the observed frequencies, shown superimposed with a kernel density estimate. Each algorithm tested (ABC, GA and NMMSO) is shown superimposed on the approximation obtained from the LHS. In each case, the distributions consist of N=512 points and the model was calibrated by varying 2 parameters (A and a) within their bounded constraints, with other model parameters fixed to their typical values (see Table 1 in Manuscript). Here, the full nonlinear model was used to simulate the model dynamics and compare the PSD to data. C) JSD between the parameter distributions obtained from the LHS and the parameter distributions obtained from each method (log scale), for subject 1. D) The number of function evaluations used to execute each method, for subject 1.

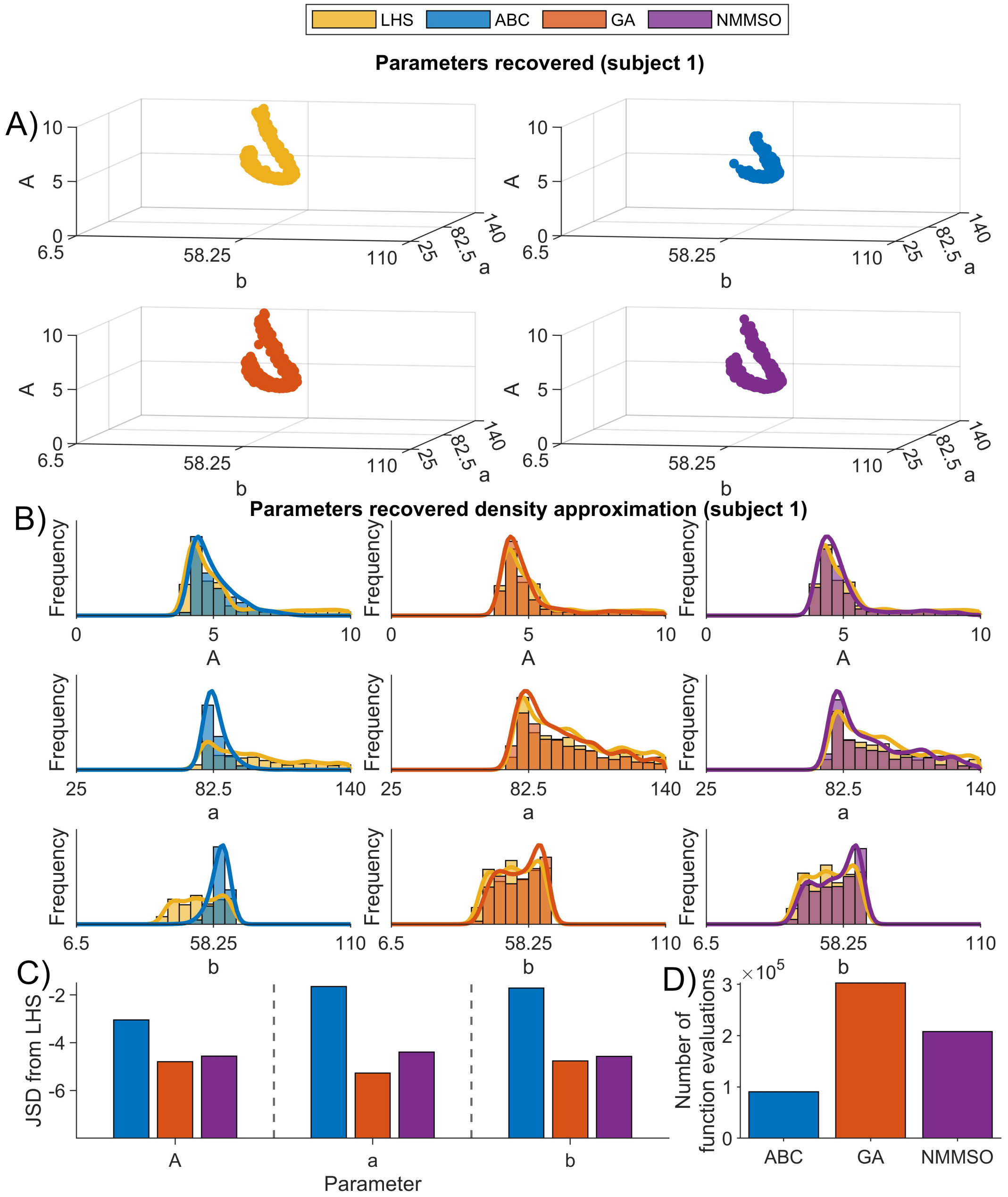

Figure S13: **Comparison of algorithms for model calibration in 3 dimensions using the full nonlinear model.** A) Model parameters recovered from the LHS (top left), ABC (top right), GA (bottom left) and NMMSO (bottom right) for subject 1. Colours indicate regions where feasible parameters were found. B) Marginal density approximations obtained using each algorithm to recover parameters from subject 1. For each parameter, the x-axis limits are set to the parameter's bounds, and the histograms give the observed frequencies, shown superimposed with a kernel density estimate. Each algorithm tested (ABC, GA and NMMSO) is shown superimposed on the approximation obtained from the LHS. In each case, the distributions consist of N=512 points and the model was calibrated by varying 3 parameters (A, a and b) within their bounded constraints, with other model parameters fixed to their typical values (see Table 1 in Manuscript). Here, the full nonlinear model was used to simulate the model dynamics and compare the PSD to data. C) JSD between the parameter distributions obtained from the LHS compared to the parameter distributions obtained from each method (log scale), for subject 1. D) The number of function evaluations used to execute each method, for subject 1.

Bayesian optimisation for likelihood-free inference

To test an additional likelihood-free inference algorithm, Bayesian optimization for likelihood-free inference (BOLFI) was implemented on subject 1 in 3 dimensions (Lintusaari et al., 2018). The results are presented in Fig S14. The ELFI python library was used to implement BOLFI, with 2000 initial evidence simulations drawn from a uniform prior, and then repeated sampling from the posterior until 512 simulations under the tolerance threshold was obtained. This initial evidence size was chosen so that the computational time to simulate BOLFI was approximately equivalent to the simulation time taken by the GA for this subject. All other hyperparameters were set to the default values implemented in the ELFI package.

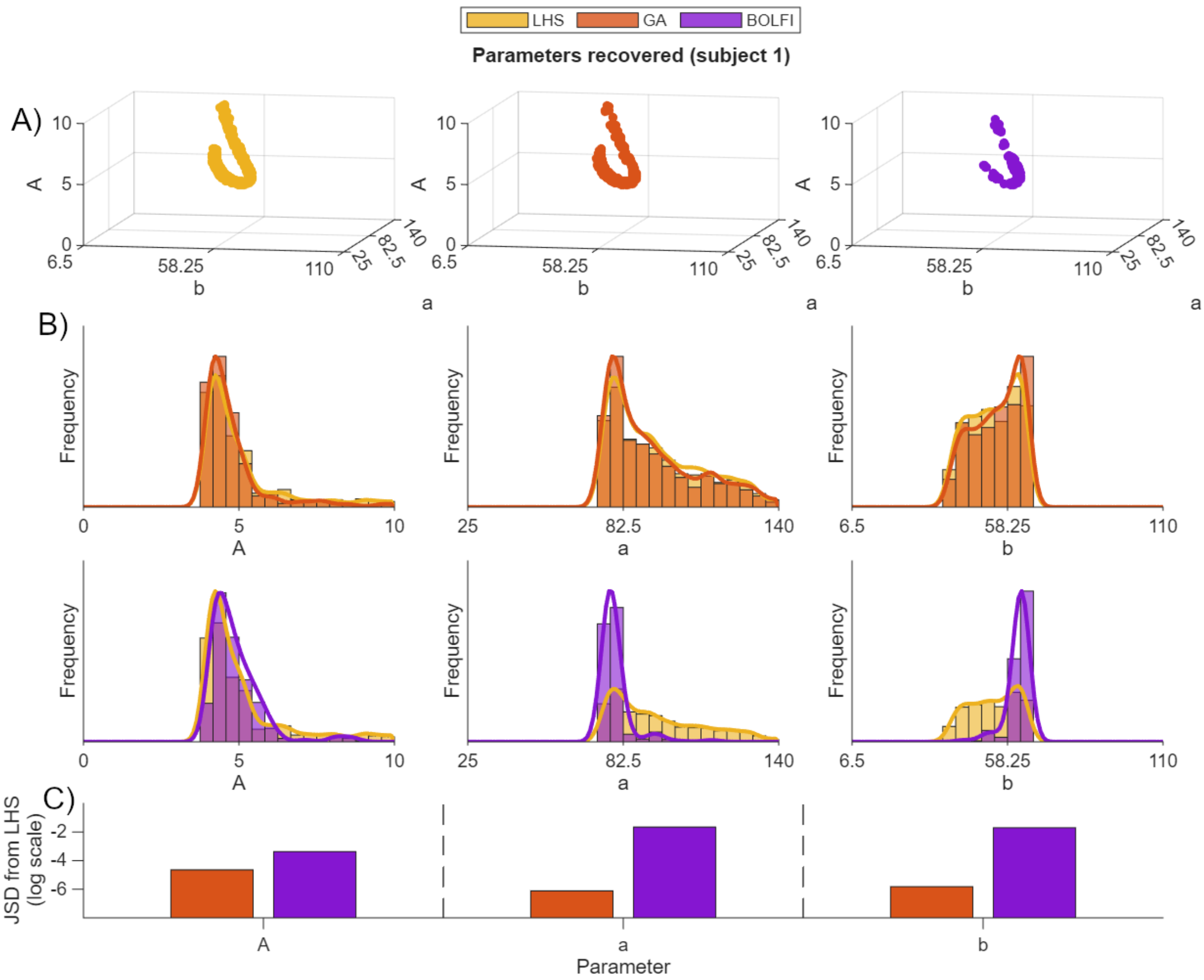

Figure S14: **Comparison of BOLFI and GA for model calibration in 3 dimensions.** A) Model parameters recovered from the LHS (top left), BOLFI (top middle) and GA (top right) for subject 1. Colours indicate regions where feasible parameters were found. B) Marginal density approximations obtained using each algorithm to recover parameters from subject 1. For each parameter, the x-axis limits are set to the parameter's bounds, and the histograms give the observed frequencies, shown superimposed with a kernel density estimate. Each algorithm tested (BOLFI and GA) is shown superimposed on the approximation obtained from the LHS. In each case, the distributions consist of N=512 points and the model was calibrated by varying 3 parameters (A, a and b) within their bounded constraints, with other model parameters fixed to their typical values (see Table 1 in Manuscript). C) JSD between the parameter distributions obtained from the LHS compared to the parameter distributions obtained from each method (log scale), for subject 1.

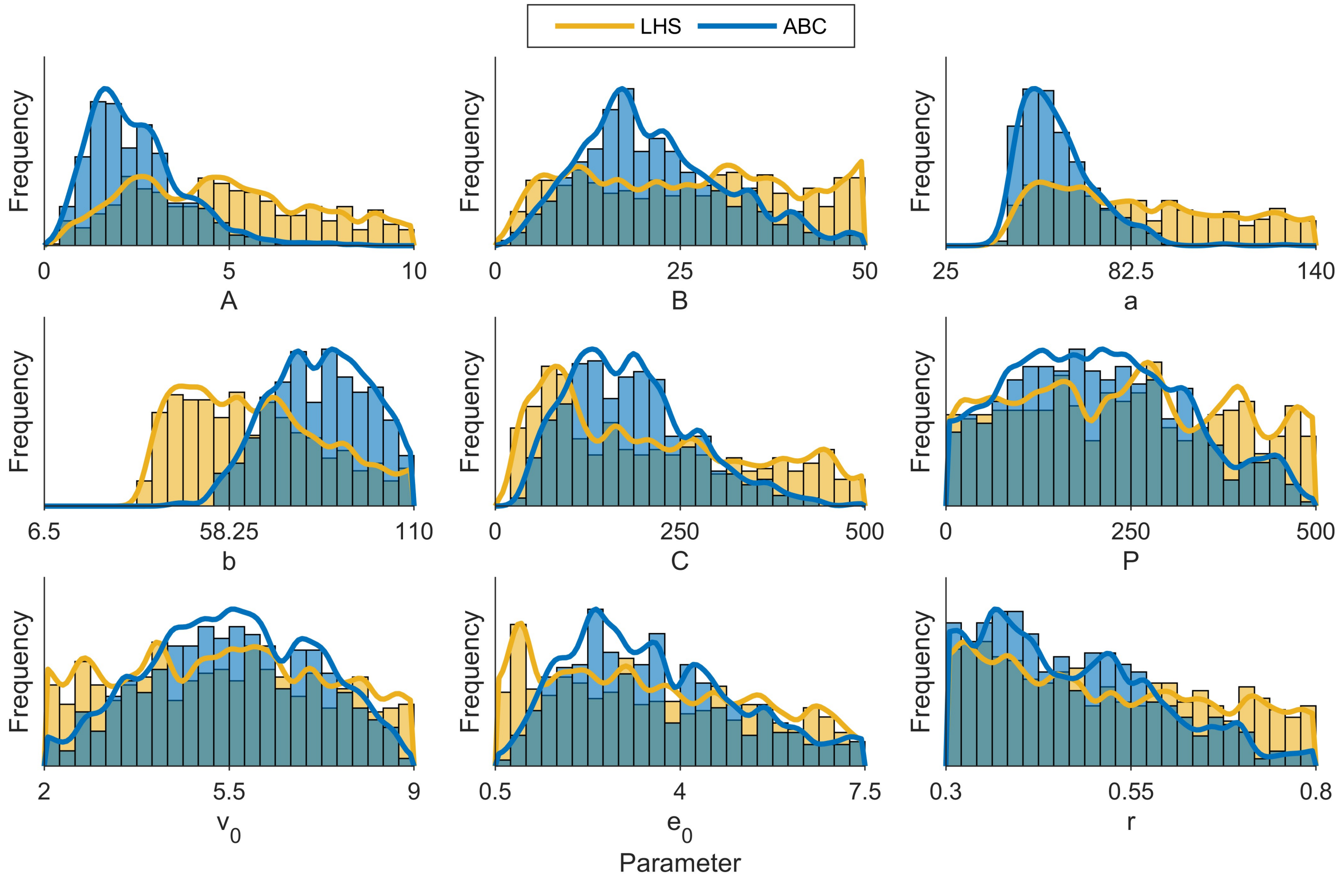

Figure S15: **Marginal parameter density approximation for subject 1, obtained using ABC in 9-dimensions.** The distributions obtained from ABC are shown superimposed on the parameter distributions obtained from LHS (see legend). For each parameter, the x-axis limits are set to the parameter's bounds, and the histograms give the observed frequencies, shown superimposed with a kernel density estimate.

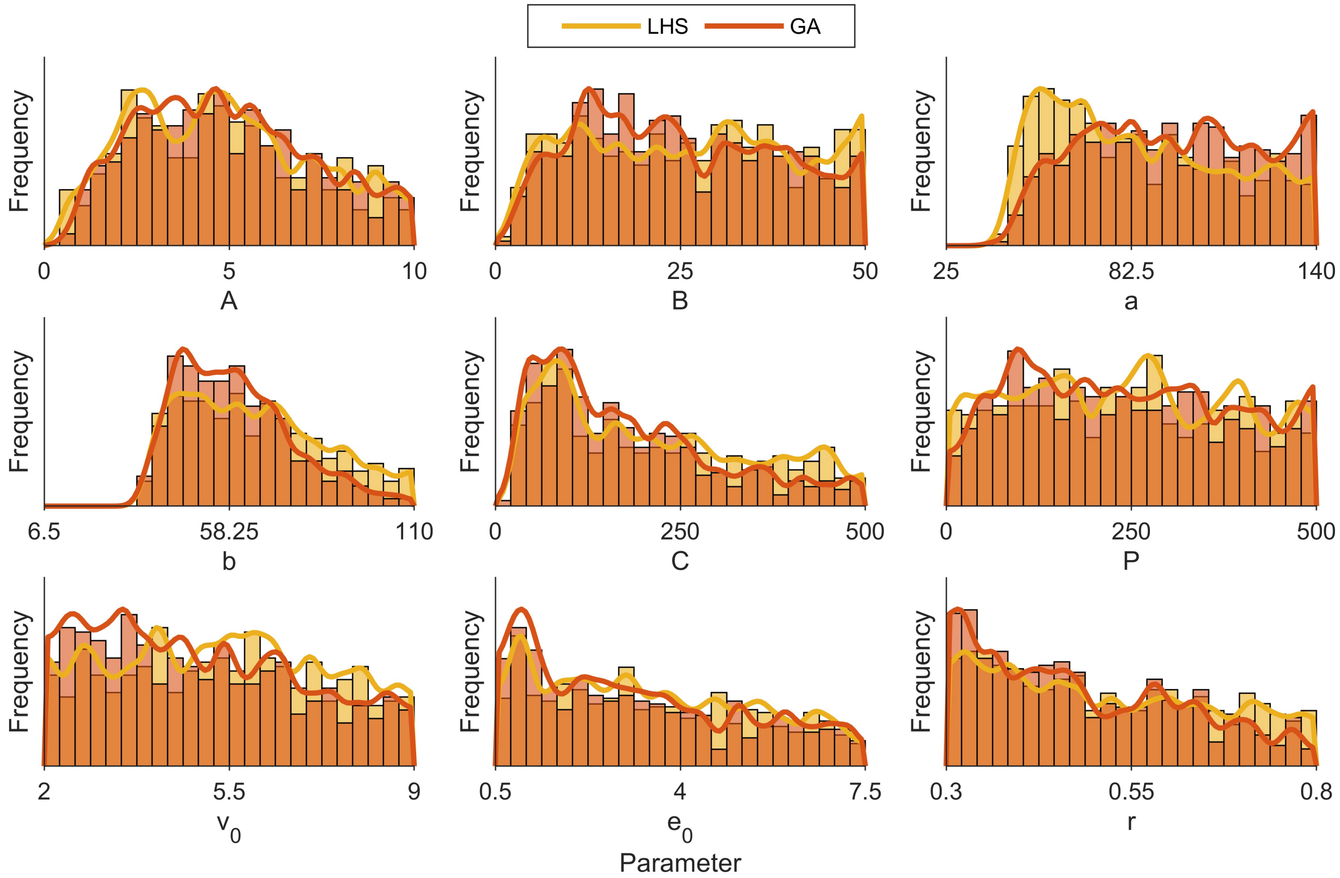

Figure S16: **Marginal parameter density approximation for subject 1, obtained using GA in 9-dimensions.** The distributions obtained from GA are shown superimposed on the parameter distributions obtained from LHS (see legend). For each parameter, the x-axis limits are set to the parameter's bounds, and the histograms give the observed frequencies, shown superimposed with a kernel density estimate.

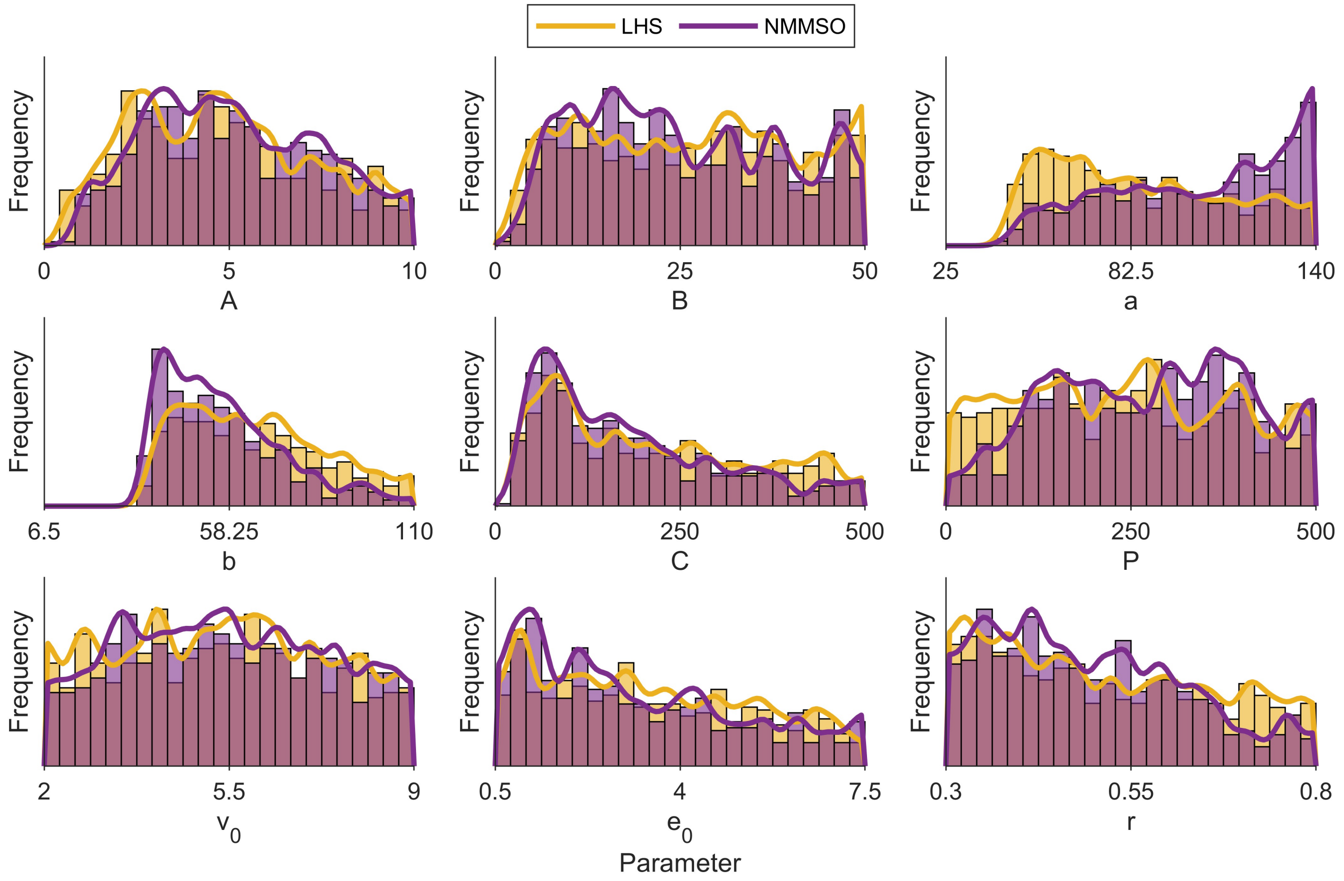

Figure S17: **Marginal parameter density approximation for subject 1, obtained using NMMSO in 9-dimensions.** The distributions obtained from NMMSO are shown superimposed on the parameter distributions obtained from LHS (see legend). For each parameter, the x-axis limits are set to the parameter's bounds, and the histograms give the observed frequencies, shown superimposed with a kernel density estimate.

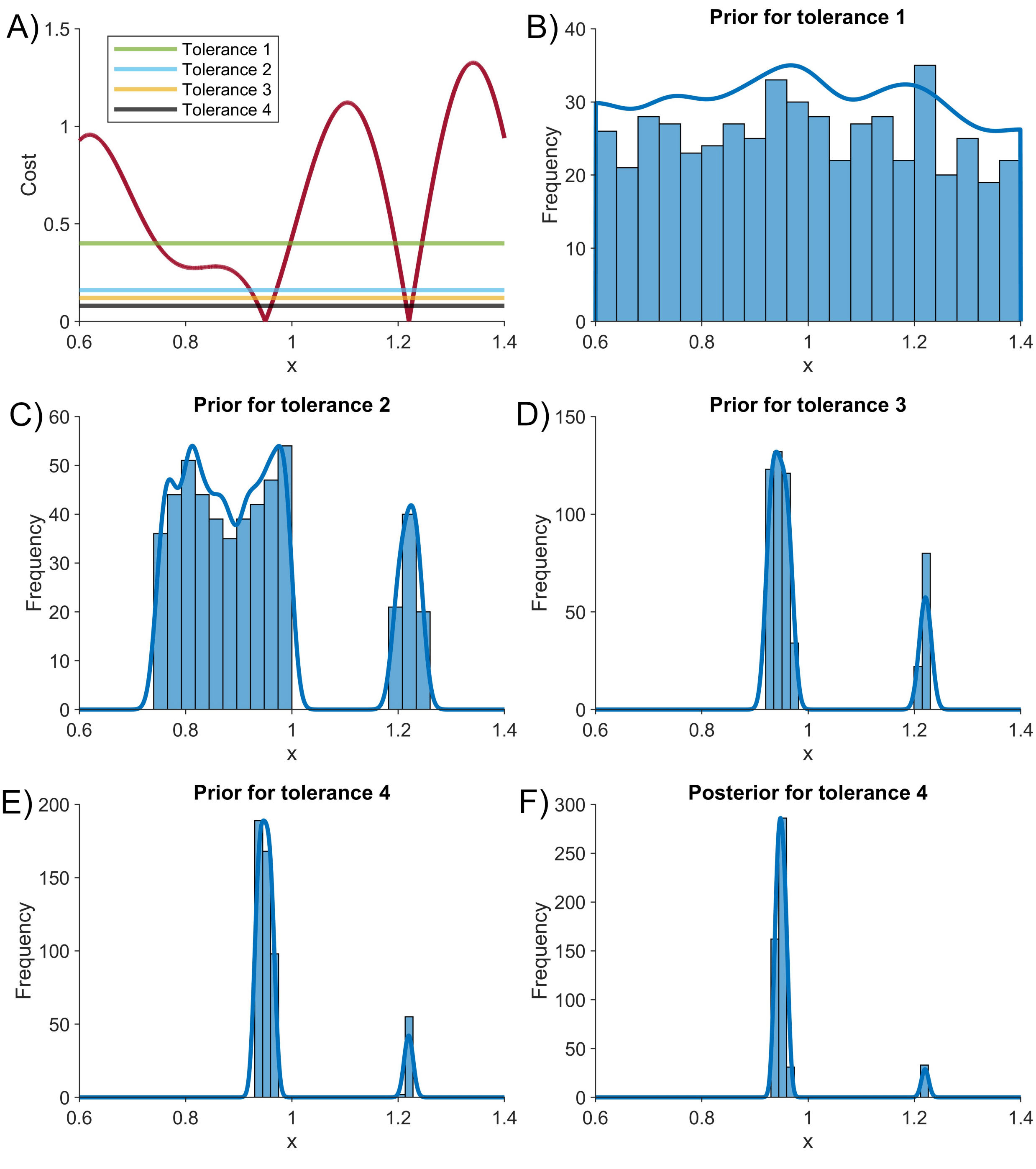

Figure S18: **Tolerance schedule of ABC on one-dimensional cost function.** A) One-dimensional cost function (see Methods in Manuscript for definition). A horizontal line representing the cost for each tolerance used in the schedule has been added. Tolerance 4 represents the final cost tolerance threshold value. B-E) Iterative priors used for each of the cost tolerances (see subtitles). Note, the first prior involves taking draws from a uniform distribution between the box constraints. The x-axis limits are set to the parameter's bounds, and the histograms give the observed frequencies, shown superimposed with a kernel density estimate. F) Posterior from the final tolerance. This posterior deviates substantially from that obtained using the LHS (see Manuscript). The ABC method used does not provide a robust inference due to overestimating density around the lower region of low cost, as a consequence of biasing the priors around this region.

References

Lintusaari, J., Vuollekoski, H., Kangasrääsiö, A., Skytén, K., Järvenpää, M., Marttinen, P., Gutmann, M. U., Vehtari, A., Corander, J., & Kaski, S. (2018). Elfi: Engine for likelihood-free inference. *J. Mach. Learn. Res.*, *19*(16), 1–7. http://jmlr.org/papers/v19/17-374.html

Welch, P. D. (1967). The use of fast fourier transform for the estimation of power spectra: A method based on time averaging over short, modified periodograms. *IEEE Trans. Audio Electroacoustics*, *15*(2), 70–73. https://doi.org/10.1109/TAU.1967.1161901
